# Earthquake-Related Changes in Species Spatial Niche Overlaps in Spring Communities

**DOI:** 10.1038/s41598-017-00592-z

**Published:** 2017-03-27

**Authors:** Simone Fattorini, Paola Lombardo, Barbara Fiasca, Alessia Di Cioccio, Tiziana Di Lorenzo, Diana M. P. Galassi

**Affiliations:** 1 Department of Life, Health and Environmental Sciences, University of L’Aquila, L’Aquila, Italy; 2 CE3C – Centre for Ecology, Evolution and Environmental Changes/Azorean Biodiversity Group and University of Azores, Angra do Heroísmo, Portugal; 3 Limno Consulting, Rome, Italy; 4 Istituto per lo Studio degli Ecosistemi, ISE-CNR, Sesto Fiorentino, Florence, Italy

## Abstract

Species interactions between stygobites (obligate groundwater organisms) are poorly known, reflecting the difficulty in studying such organisms in their natural environments. Some insight can be gained from the study of the spatial variability in microcrustacean communities in groundwater-fed springs. Earthquakes can increase hydraulic conductivity in the recharge area of karstic aquifers and flow rates in discharge zones, thus dislodging stygobites from their original habitats to the spring outlets. Earthquakes are expected to alter species spatial niche overlap at the spring outlets, where stygobites coexist with non-stygobites living in benthic and subsurface habitats. We compared the abundance of stygobiotic and non-stygobiotic microcrustaceans in groundwater-fed springs before and after the 6.3-M_w_ earthquake that hit the karstic Gran Sasso Aquifer (Italy) in 2009. Pre-seismic (1997, 2005) overall niche overlaps were not different from null expectations, while post-seismic (2012) species mean niche overlaps were higher, following the redistribution of animals caused by the earthquake-triggered discharge. The reduced abundance of stygobites following their dislodgement from the aquifer and the concomitant displacement of non-stygobites led to a higher post-seismic co-occurrence of stygobites and non-stygobites. Changes in aquifer structure destroyed pre-seismic species segregation patterns by creating new or strengthening already existing interactions.

## Introduction

The quantitative study of niche overlap in community ecology dates back to the pioneering work of MacArthur and Levins^1^, who introduced an index based on the relative utilisation of different segments of a niche resource axis (dimension). This work paved the way for a fruitful research line on niche theory especially in the 1970s, including the possible relationships of niche overlap with competition, fitness, diversity and community structure^2–5^.

According to classical ecological theory^6, 7^, a differential utilisation of resources, i.e. low niche overlap, is considered essential for the coexistence of sympatric species. Because of the variety of dimensions that form species’ niches^7^, overlap on one resource axis may indicate diversification along other resource axes^2, 5, 8^, which may explain the coexistence of species greatly overlapping along one or a few dimensions. Microhabitat, diet, and temporal activity (e.g. seasonal or diel differences between species in habitat exploitation) are the three most frequently used characteristics to measure niche overlap^2, 5, 9^. In particular, interspecific overlap in habitat use has been invoked as a key factor in shaping ecological communities from the small (e.g., close-range interspecific interactions^10^) to the large scale (e.g., species distribution areas, including evolutionary aspects^11^).

Groundwater animal communities comprise species that have developed adaptive traits to survive in total darkness, lack of photosynthesis, low food availability, and strict dependence on the organic matter entering groundwater from the surface, potentially leading to severe inter- and intraspecific competition^12^. However, detailed data about how groundwater species use space at fine scale are lacking, especially because sampling is hampered by the limited access to groundwater. Thus, most of the available information on groundwater species ecology is based on sampling groundwater-fed springs, caves and boreholes^13, 14^.

Karstic aquifers are complex systems whose parts are interconnected by water that flows from surface recharge areas to the aquifer outlets, such as springs. This interconnection between the recharge area and the different parts of the aquifer should promote faunal homogenisation by favouring species drift, especially for non-stygobites that live on the surface, enter the aquifer through fast infiltration pathways and are dispersed by groundwater flows, especially during high-discharge periods^15, 16^. These species also reach the spring environment via the surface water hydrological continuum. By contrast, obligate groundwater species (i.e. stygobites) typically show restricted and idiosyncratic distributions, indicative of limited dispersal abilities^14^.

The very high level of stygobiotic endemism, with more than 90% of the groundwater fauna comprised of local endemics^17^, also supports the view of low dispersal for most groundwater species (but see ref.^18^). This is consistent with the fact that animal communities inhabiting groundwater environments are less subject to strong environmental variations than their counterparts that live on the surface. However, groundwater environments can be altered by major disturbance events such as earthquakes^14, 19^.

The L’Aquila earthquake on 6 April 2009 (6.3 M_w_) changed the groundwater flow of the Gran Sasso Aquifer (GSA) and consequently of the Tirino River valley, where ~65% of the aquifer discharge is located^20^. The earthquake induced an increase in the bulk hydraulic conductivity of the recharge area, near the ruptured fault zone (attributable to fracture clearing and/or microcrack formations), and led to an anomalous rising of the water table and flow rate in discharge zones^21^. As a result, the overall physiography of the spring system changed from rheo-limnocrene to predominantly limnocrene^22, 23^. These changes altered the community organisation of subsurface (i.e. below the spring bed) microcrustaceans at the GSA main outlet at the Tirino Springs (TS), especially via a strong reduction in the abundance of stygobites^14^.

Because of the tight relationships between ground shaking, aquifer strain, fracturing and fracture clearing and changes in stygobites’ habitats, we expected an impact of the 2009 earthquake on species distribution in the aquifer and at the major aquifer discharge points. In particular, the post-seismic lower densities and patchier occurrence of stygobites at the TS, coupled with the persistent diffuse occurrence of non-stygobites^14^, could have increased species coexistence by reducing the degree of spatial niche overlap among species.

To test if the earthquake increased interspecific spatial overlap in spring communities, we compared species co-occurrence in low-discharge (1997), high-discharge (2005), and post-seismic, very high-discharge (2012) hydrological years using the Copepoda (Crustacea) as a model biotic group. Copepods also comprise ~80% of the total meiofauna density at TS^14, 22, 23^. In particular, we tested if the earthquake-induced higher discharge increased the niche overlap of copepod species by reshaping their micro-distribution in the TS.

## Results

A total of 22 species, 9 of which (~40%) are stygobites, were found in the three sampling years (S1 Supporting Information). Total number of individuals retrieved from samples ranged from 992 (average number of individuals per sample ± SE = 124.000 ± 26.012) in 1997 (a low-discharge year) to 2750 (average number of individuals per sample ± SE = 343.750 ± 118.583) in 2005 (an above-average discharge year), and then back to 910 (average number of individuals per sample ± SE = 113.750 ± 49.846) in post-seismic 2012 (a very high discharge year, Table S1). Average number of individuals per sample did not differ significantly between years (ANOVA: *F*_2,21_ = 2.941, *P* = 0.075). Stygobiotic species contribution to total abundance decreased from 59% in 1997 to 35% in 2005, then further crashed to 21% in 2012 (contingency table: χ^2^ = 310.015, df = 2, *P* < 0.0001). Despite a marked reduction in total abundance after the 2009 earthquake, the harpacticoid copepod *Nitocrella pescei* remained the most common stygobite throughout the sampling period (S1 Supporting Information).

Overall mean niche overlap was 30% in 1997, 34% in 2005 and 58% in 2012 (Table 1). Mean overall niche overlaps in 1997 were not significantly higher than the respective simulated assemblages for all three types of assemblages (whole assemblage, stygobites only and non-stygobites only), regardless of the randomisation algorithm used (*P* > 0.05 in all cases; Table 1). Mean overall niche overlap in 2005 was significantly higher than the simulated assemblages constructed using the RA3 randomisation algorithm (*P* < 0.01) but not under the assumption of the RA2 algorithm (*P* = 1.000) (Table 1). The mean niche overlap of stygobiotic species in 2005 was not significantly higher than expected regardless of the randomisation algorithm used (*P* > 0.05 in all cases; Table 1). The mean niche overlap of non-stygobites was significantly higher than expected under RA3 (*P* = 0.001), but not under RA2 (*P* = 0.461) (Table 1). Finally, in 2012, mean overall niche overlap for the whole assemblage, stygobiotic mean overall niche overlap, and non-stygobiotic mean overall niche overlap were all significantly higher than expected, regardless of the randomisation algorithm used (*P* < 0.005 in all cases; Table 1).

**Table 1:** Community-level interspecific overlap between spring subsurface copepods.

	Observed overlap	RA2		RA3	
		Expected overlap ± variance	*P* (observed ≥ expected)	Expected overlap ± variance	*P* (observed ≥ expected)
1997					
All species	0.296	0.296 ± 0.000	0.999	0.263 ± 0.000	0.065
Stygobites	0.267	0.317 ± 0.001	0.945	0.262 ± 0.002	0.411
Non-stygobites	0.321	0.391 ± 0.001	0.994	0.265 ± 0.001	0.077
2005					
All species	0.336	0.402 ± 0.000	1.000	0.281 ± 0.0003	0.009
Stygobites	0.208	0.312 ± 0.001	0.999	0.249 ± 0.003	0.776
Non-stygobites	0.456	0.452 ± 0.001	0.461	0.303 ± 0.001	0.001
2012					
All species	0.584	0.500 ± 0.001	0.005	0.316 ± 0.000	< 0.0001
Stygobites	0.453	0.377 ± 0.001	0.016	0.335 ± 0.003	0.028
Non-stygobites	0.695	0.567 ± 0.003	0.007	0.302 ± 0.001	< 0.0001

Observed Pianka’s niche overlap index within communities of subsurface copepods at the Tirino Springs (Abruzzo, Central Italy), and expected overlap for null assemblages, before (1997 and 2005) and after (2012) the earthquake. RA2 (relaxed niche breadth) and RA3 (retained niche breadth) are the two types of algorithms used to construct null-assemblages.

Using pairwise values (*O*), niche overlap varied significantly among years and type of species pairs (stygobite–stygobite or s,s; non-stygobite–non-stygobite or n,n; or stygobite–non-stygobite or s,n) (Table 2). Bonferroni’s tests indicate significant differences in the mean *O* values among all years (*P*_1997–2005_ = 0.026, *P*_1997–2012_ < 0.00001, *P*_2005–2012_ < 0.00001), with mean values increasing from 1997 to 2005 and again from 2005 to 2012. The Bonferroni tests indicate that the mean *O* values of pairs in which both species were non-stygobites (n,n) were significantly higher than those in which both species were stygobites (s,s) (*P* < 0.00001) or in which one species was a stygobite and the other one was not (s,n) (*P* < 0.00001); mean *O* values of s,s and s,n pairs were statistically similar (*P* = 0.240).

**Table 2:** ANOVA for overlap indices between species pairs (*O*).

	SS	df	MS	*F*	*P*
Type of pairs	3.020	2	1.510	12.107	< 0.0001
Error	18.706	150	0.125		
Year	4.258	2	2.129	36.375	< 0.0001
Year × type of pair	0.568	4	0.142	2.427	0.048
Error	17.561	300	0.059		

Type of pairs refers to the following classification: (i) both species in the pair are stygobites, (ii) one species is stygobite and other one is non-stygobite, and (iii) both species are non-stygobites. Year refers to the three compared years: 1997, 2005 and 2012.

**Figure 1:**
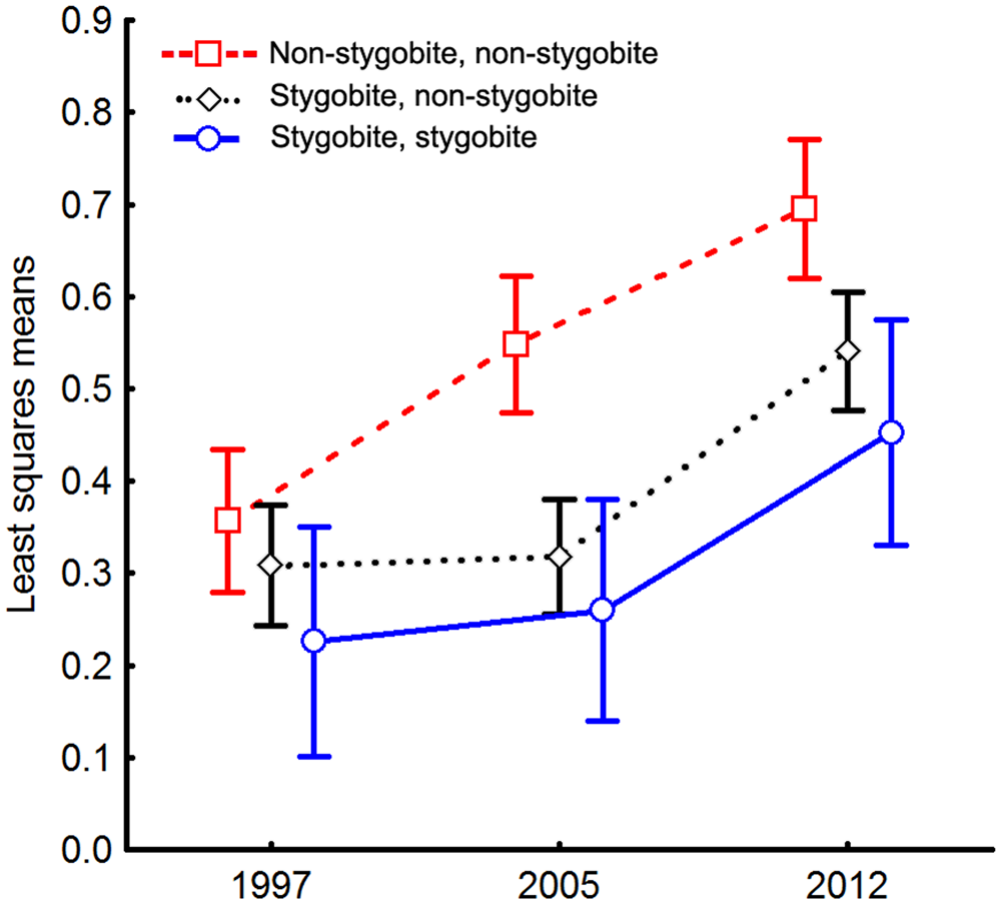
Between-species niche overlap in time. Interaction plot for variation in niche overlap between species pairs (*O* values) between years. Circles (and continuous line) indicate species pairs in which both species are stygobites; diamonds (and dotted line) indicate species pairs in which one species is a stygobite and other one is a non-stygobite. Squares (and broken line) indicate species pairs in which both species are non-stygobites.

The interaction term was marginally significant (*P = *0.048, Table 2), which suggests that the different pair types responded differently in the three years. Inspection of the interaction plot (Fig. 1) and use of paired *t*-tests indicate that s,s and s,n pairs had similar mean values of niche overlap between 1997 and 2005 (*P*_s,s_ = 0.672, *P*_s,n_ = 0.833), with a significant increase between 2005 and 2012 (*P*_s,s_ = 0.024, *P*_s,n_ < 0.0001), whereas the spatial overlap for n,n pairs increased significantly between 1997 and 2005 and again between 2005 and 2012 (*P*_n,n_ < 0.0001 in both cases).

**Figure 2:**
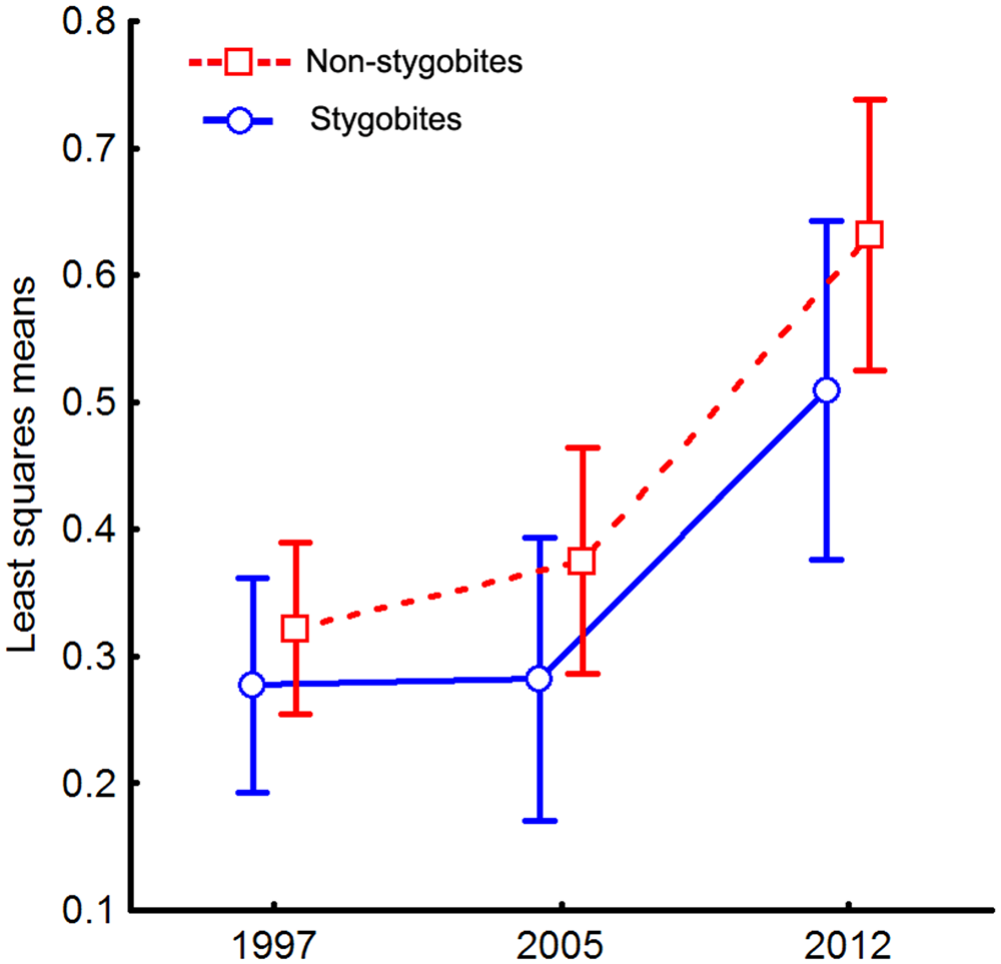
Average species niche overlap in time. Interaction plot for variation in average species niche overlap (*O*_sp_ values) between years. Circles (and continuous line) indicate stygobites; squares (and broken line) indicate non-stygobites.

Using species mean overlap values (*O*_sp_), overlap varied significantly between years regardless of species habitat specialisation (stygobites vs non-stygobites) (Table 3). Bonferroni’s tests indicate that the 2012 *O*_sp_ values were significantly higher (*P* < 0.00001 for both the 1997–2012 and the 2005–2012 comparisons), but the difference between 1997 and 2005 was not significant (*P* = 0.870) (Fig. 2).

**Table 3:** ANOVA for average species niche overlaps (*O*_sp_).

	SS	df	MS	*F*	*P*
Specialisation	0.096	1	0.096	2.490	0.134
Error	0.616	16	0.039		
Year	0.757	2	0.378	38.287	< 0.0001
Year × Specialisation	0.013	2	0.007	0.663	0.522
Error	0.316	32	0.010		

Specialisation indicates whether a species is a stygobite or a non-stygobite. Year refers to the three compared years: pre-seismic (1997, 2005) and post-seismic (2012).

An analysis of co-occurrence (C-scores) revealed that increases in niche overlap were paralleled by a reduction in species segregation. C-scores decreased from 1997 (C = 1.948) to 2005 (C = 1.457) to 2012 (C = 0.931), which means that the randomness of species distribution increased. A repeated-measures ANOVA (rANOVA) showed that C-scores varied significantly among years (SS = 91.460, MS = 45.730, *F*_2,300_ = 12.661, *P* < 0.00001) but not for type of species pairs (SS = 0.729, MS = 0.364, *F*_2,150_ = 0.008, *P* = 0.9254) and the species × year interaction was not significant (SS = 28.675, MS = 7.169, *F*_4,300_ = 1.985, *P* = 0.097).

## Discussion

In general, mean species niche overlaps were significantly higher in 2012 than in 1997 and 2005, for both stygobites and non-stygobites. Although results for the 2005 community are unclear (because the two algorithms produced contrasting outputs), results for 2012 clearly indicate a high degree of niche overlap. However, species pair interactions differed significantly also between 1997 and 2005, with changes in niche overlap that varied according to the type of species pair. Namely, pairs in which both species were non-stygobites increased their niche overlap significantly from year to year, whereas the niche overlap for pairs in which one or both species were stygobites increased significantly only in 2012. These results suggest that while non-stygobites may tune their utilisation of the space in response to hydrological changes, stygobites tend to be less adaptable, as expected in organisms specialised to live in genuine groundwater habitats. Only a major disruptive event like an earthquake seems to force a strong niche overlap among stygobites. Though this hypothesis awaits empirical confirmation, it is in line with the earthquake-induced flushing of stygobiotic species and their abundance changes discussed elsewhere^14^.

It has been recently suggested that springs may be considered as islands for stygobites^24^. According to an insular model, the copepod assemblages of the TS should form a metacommunity regulated by patch dynamics models. In general, four metacommunity models have been proposed to account for variation in local community structure: (1) neutral model (species are essentially equivalent in their competitive and dispersal abilities, and the metacommunity is mainly regulated by stochastic immigration and extinction processes), (2) species sorting (individual species respond differently to environmental heterogeneity), (3) mass effects (dispersal and environmental heterogeneity interact to determine species abundance and composition), and (4) patch dynamics (interacting species differ from each other in being either good competitors or good colonizers within a uniform environment), but the last two perspectives – mass effects and patch dynamics – have been recently suggested to be special cases of the species sorting perspective^25, 26^. Significant variations in niche spatial overlaps determined by strong environmental changes support the idea that the TS copepods are a metacommunity regulated by the interactions of mass effects and patch dynamics.

Our results indicate that the TS subsurface copepod community is not only subjected to wide fluctuations in species abundances as a consequence of relatively small environmental changes, but also to variations in niche overlap between species pairs determined by anomalous high disturbance events. The term “disturbance” has a variety of meanings including any event that may kill or displace organisms, deplete consumable resources (e.g., living space and food) and degrade or destroy habitat structure^27^. Spring communities constantly experience small variations in discharge, such as the increase observed in 2005 at TS. However, the stygobiotic species assemblages of the TS system were not affected by this small variation. This is because the TS stygobiotic species primarily reside in low-flow “chambers” located inside the carbonate massif feeding the spring outlets^14^. With the high increase in discharge due to the main shock, the water pressure in the fast-flowing conduits of the aquifer decreased, allowing the microcrustaceans of the storage subsystem to be flushed out, enter into the fast-flowing conduits and then into the main aquifer outlets, i.e. the Tirino Springs, where they became stranded into the springbed sediments, thus co-occurring with the non-stygobiotic fauna that normally lives there. This explains the absence of a significant difference in niche overlap between 1997 and 2005 for s,s pairs. TS non-stygobites were negatively affected by 2005 discharge variations. Some stenotopic non-stygobiotic species might have left the suddenly inhospitable microhabitats, migrating into other microhabitats. This pattern was revealed by the increase in spatial overlap for n,n pairs, paralleled by a reduction in species segregation. However, a certain species *a* can simultaneously increase its habitat overlap with species *b* and reduce its overlap with species *c*, so that its overall niche overlap with the other species does not change, as indicated by the lack of differences in *O*_sp_ values between 1997 and 2005 for s,n pairs. Thus, *O* values can be subjected to high variation even when the mean interaction of a species with all the others taken together remains similar. This is reasonable also in terms of competition. It is difficult to study interspecific competition empirically^10^. Based on earlier theoretical and mathematical considerations^28^ and the assumptions of niche partitioning by fluctuation-dependent mechanisms of coexistence^29^, it follows logically that if a species *a* has two competitors, say *b* and *c*, when *b* is more abundant than *c*, *a* will compete more with *b* than with *c*, but if *c* becomes more abundant than *b*, then *a* will begin to compete more with *c* and less with *b*; yet the mean competition of *a* with *b* and *c* might remain the same. This type of multiple changes in the interaction intensity between species (i.e. in *O* values) may explain the lack of differences in *O*_sp_ values between 1997 and 2005 for s,n pairs.

However, the 2012 situation reveals a completely different scenario, because mean s,s and s,n overlaps increased significantly in 2012 after no observable change between 1997 and 2005. Therefore, moderate differences in aquifer discharge seem insufficient to drive substantial changes in species relative abundances such as those observed in 2012, which can be associated with the earthquake-induced exceptionally high aquifer discharge, at least for s,s and s,n pairs. Such a strong earthquake induced aquifer dewatering of the otherwise low-flow “chambers” where stygobites resided^14^. The intensity of this disturbance was also sufficient to redistribute the pre-seismic stygobiotic species, indicating that background variations in aquifer discharge during non-earthquake years (1997 to 2005) did not influence the spatial arrangements of obligate groundwater copepods at the main aquifer outlets appreciably, while a catastrophic event like a 6.3-M_w_ earthquake did (1997/2005 to 2012). Increases in spatial overlap were paralleled by a reduction in species segregation (C-score analysis), indicative of major species displacement. This suggests that the increase in spatial overlap within s,s and s,n pairs is related to an increase in interspecific interactions in turn determined by changes in species distribution in the spring subsurface microhabitats. Shifts from segregation to aggregation/randomness have also been observed in ant communities suddenly altered by the introduction of non-native species^30, 31^ and severe, albeit transient, changes were noticed in lacustrine planktonic communities after an earthquake^32^, suggesting that this pattern can be a recurrent consequence of a strong disturbance.

Total copepod abundance at TS remained low three years after the 2009 earthquake^14^, which may be a consequence of two concurrent events: (1) most stygobites living in the storage subsystems of the GSA were flushed out during the mainshock, strongly reducing the populations in the primary habitat, thus preventing subsequent recolonisation of the GSA outlets; (2) the strong increase in aquifer discharge at the TS increased the water flow at the main spring outlets, favouring a severe rearrangement of the spring habitats by changing springbed areas of erosion and deposition of sediments, together with sediment composition across spring sites, thus redistributing also the meiofauna living among sediment particles, and hence leading to larger values of spatial overlap^33, 34^. As a consequence of these changes, non-stygobites probably exerted high competitive pressures over stygobites in TS, a secondary habitat for stygobites, which are highly specialised on stable aquifer conditions, and cannot reproduce into the spring, although they can survive there for possibly 3 or 4 years^35, 36^. Abundances of stygobiotic species were thus further decreased by non-stygobites. Of course, not all species responded in the same way. For example, the dramatic post-seismic reduction in the overall abundance of the three most abundant stygobites (*Nitocrella pescei*, *Diacyclops paolae*, and *Parastenocaris lorenzae*) allowed the fourth-abundant stygobiotic *Elaphoidella mabelae* to maintain its pre-seismic densities because of the latter’s wider ecological plasticity, this species having a habitat preference similar to that of both *Diacyclops paolae* and *Parastenocaris lorenzae* ^22^, a condition that likely favoured spatial niche replacement by the ecologically tolerant *E. mabelae*. Similarly, the post-seismic crash in the abundances of the most dominant stygobites may have allowed the non-stygobiotic *Attheyella crassa* to increase its relative abundance.

Very fine sand and particulate organic matter (POM) significantly increased in 2012 at the TS outlets^14^. The “niche-overlap hypothesis” predicts increased separation of niches within a community as resource levels decrease^2, 5, 37^. Thus, increased resource availability (POM, an important food source for groundwater fauna) would promote higher niche overlap, contributing to the observed post-seismic increase in mean interspecific interaction strength.

The spatial redistribution determined by ground shaking and the steep increase in discharge altered the overall physiography of the spring system, increased the surface water velocity, and rearranged habitat patches. This led to the observed decrease in stygobiotic abundances in 2012, especially for the species that were the most abundant before the earthquake and which became spatially more aggregated at close range near the main aquifer outlets after the earthquake. The decline of the stygobiotic populations into the aquifer precluded a substantial post-seismic recolonisation of the springs, where true groundwater species (i.e., stygobites) are unable to reproduce^14^. At the same time, the displacement of non-stygobites from their original microhabitats due to seismic redistribution may have determined a higher co-occurrence of stygobiotic and non-stygobiotic species at the microhabitat level.

## Conclusions

The mainshock of the L’Aquila earthquake on 6 April 2009 dramatically altered the microcrustacean community patterns at TS, the main spring outlets of the GSA, by increasing interspecific spatial overlap (higher *O* index values) and reducing species segregation (lower C-scores). Our overall interpretation of the observed changes in niche overlap is that the earthquake substantially modified the spatial arrangements of the TS subsurface environment, thus reshaping species distributions below the springbed. Changes in species micro-distributions destroyed pre-seismic species segregation patterns by creating new or strengthening already existing interspecific interactions. Among the available metrics to detect the effects of a disturbance, the niche overlap indices used in this study proved to be appropriate, providing an added value to simple species counts. Though our analyses were useful to elucidate general patterns, we urge specific research targeting the autecology of subterranean and surface microinvertebrates to shed light on the nature and strength of their interactions.

## Methods

### Study area

The TS area is a spring complex at the boundary of the karstic Gran Sasso Aquifer (GSA) located in the Gran Sasso Massif in central Italy (Apennines mountain range), featuring the highest peak south of the Alps (Corno Grande, 2922 m a.s.l.) and characterised by a high- to moderate-altitude montane landscape with low human impact. The GSA is a fractured aquifer with fast-flowing sections (karstic conduits) and interconnected small chambers^14^. The TS is the largest GSA-fed spring system, receiving ~65% of the GSA discharge^20^. The short (~15 km) Tirino River originating from TS joins the Aterno-Pescara River before eventually emptying into the Adriatic Sea. Spring and river water quality is high until the Tirino River receives an aquaculture effluent at mid-course^38^. Mean discharge, water temperature, pH, dissolved oxygen and nitrate content at TS and along the first half of the Tirino River tend to remain relatively constant over time in the absence of major disruptions^14^.

Mean annual discharge at TS was relatively low in the first sampling year (1997: mean ± SD: 5.68 ± 0.21 m^3^s^−1^); it was slightly above-average in the second sampling year (2005: 6.02 ± 0.26 m^3^s^−1^) and was well above average in the third sampling year (2012: 7.14 ± 0.26 m^3^s^−1^) due to a 3-yr rising in discharge caused by the 6.3-M_w_ 2009 earthquake, before slowly returning to pre-seismic values in summer 2013^14^.

The whole area of the GSA aquifer (700 km^2^) was affected by the 2009 earthquake, even if the epicentre was about ~30 km far from GSA. Because of the increase in discharge occurred immediately after the mainshock^23^, the fast-flowing conduits of this fractured aquifer become emptied and water pressure decreased, allowing the groundwater (and the associated microcrustaceans) of the storage subsystem to flush out, enter the fast-flowing conduits and reach the main aquifer outlets, i.e. the Tirino Springs. Details about the study site and discharge patterns at TS are given elsewhere^14, 22, 23^.

### Sampling

Copepods were collected at eight sampling sites at the TS adopting a random sampling method with four temporal replicates per site in the following two-month periods: Jan–Feb, Mar–Apr, Jul–Aug, Sep–Oct in each year (1997, 2005 and 2012) (Supplementary Information Tables S1–3). For each two-month period, sampling was carried out between the end of the first month and the beginning of the second one.

We considered each sampled spring outlet as a “natural sampling unit” and hence as a resource state (=microhabitat space)^3^; thus, the number of individuals of each species collected from the various sampled springs was considered as a proxy for the amount of used “resource”.

Subsurface samples of water were collected from the springbed (sediment patches and karstic fractures) with a hand-made Bou-Rouch pump^39, 40^ and mobile pipes hammered at each sampling site. The Bou-Rouch sampling equipment included a 125-cm long steel standpipe with a 2-cm internal diameter and a 15-cm terminal section perforated by 10 holes (5-mm diameter). The piston pump was then manually operated at the fastest rate possible, to extract a standardised sample size of 20 L, a volume of water/sediments that is sufficient to obtain reliable estimates of abundance of rare species^41^. Each site- and temporal-specific sample was a composite of subsamples from 0.3, 0.7 and 1.5 m below the springbed to reduce the inherent variability in densities stemming from differences in the dimension of the interstitial voids in springbed sediments. When using a Bou-Rouch pump, the true volume of sampled habitat remains unknown, because the same amount of pumped water may come from different volumes of sediment. In general, this may be problematic for comparing species densities. However, the organisms investigated in this study are much more dependent on the water medium than on the characteristics of the sediments in which water is contained (and which are difficult to reconstruct). Moreover, if the pump collects the same quantity of water from different volumes of sediments due to their anisotropy, this also reflects how microcrustaceans may circulate in the interspaces within the sediment. Thus, to obtain comparable (standardised) values of species abundances, it is more important to collect the same quantity of water, rather than a standard volume of sediments.

The meiofauna was extracted by filtering the 20-L samples through a hand net (mesh size = 60 µm). Samples were preserved in 80% ethyl alcohol. Individuals were later sorted, counted, identified to species level, and assigned to two ecological categories: obligate (stygobites) or non-obligate groundwater species (non-stygobites). Stygobites are strictly dependent on groundwater to complete their life cycles, but drift or are washed periodically to the aquifer outlets following the groundwater flow. Non-stygobites are freshwater species that live on the springbed surface, or in sediment interstices (e.g., to avoid predation) or are habitat generalists. Details about sampling protocols and species identification procedures are elsewhere^14^.

### Calculation of habitat overlap and significance of mean values

To express habitat overlap between species pairs, we calculated the Pianka index of niche overlap^28^ using EcoSimR 1.00^42^:

$${O}_{jk}={O}_{kj}=\frac{\sum _{i}^{n}{p}_{ij}{p}_{ik}}{\sqrt{\sum _{i}^{n}{p}_{ij}^{2}\sum _{i}^{n}{p}_{ik}^{2}}}$$

where *O*_*jk*_ is Pianka’s index of niche overlap of species *j* over species *k*, *O*_*jk*_ is the reciprocal overlap of *k* over *j*, *p*_*ij*_ is the proportion of the *i* ^th^ resource used by species *j*, *p*_*ik*_ is the proportion of the *i* ^th^ resource used by species *k*, and *n* is the total number of resources. The *O*_*jk*_ index is a symmetrical elaboration of Levins’s^28^ own original *α* index of niche overlap. Conceptually, both formulations are correlations between species distributions over discrete resources (e.g., food types or spatial patches). However, Levins’s *α* index is asymmetrical (with the overlap of species *j* over species *k* not necessarily equal to the reciprocal overlap of *k* over *j*) and may be >1, depending on resource use of the two species, whereas the *O*_*jk*_ index is symmetrical and ranges between zero (absence of overlap) and one (complete, maximum possible overlap). Following criticism^43^ of the extrapolation of competitive interactions from overlap indices, more modern analyses of niche overlap are meant as descriptions of interspecific interactions *sensu lato*, unless competitive interactions are known *a priori* ^10^. The asymmetrical nature of Levins’s *α* index is applied when a high detail of interspecific interactions is sought or known *a priori* ^10, 44^. Pianka^’^s *O*_*jk*_ index instead is preferred when species autecology is not known in sufficient detail, when a standardised index is desired (as 0 ≤ *O*_*jk*_ ≤ 1), and/or when analyses of general patterns rather than hyperdetailed interactions are sought^37, 45^. Given the poorly known autecology of groundwater fauna^14^, and our desire to provide basic community-level information that may be used later in detailed autecological studies, we have opted for Pianka’s *O*_*jk*_ index rather than Levins’s *α* index. Following the current views in ecology, we have not automatically implied competition from *O*_*jk*_ values, though negative (competitive-like) mutual influences of two coexisting species may be suspected when overlap over key resources is high^5^, and there is evidence of interspecific competition in cave microcrustaceans^46^. A detailed review of the conceptual background of Levins’s *α* index and of the overlap ≠ competition argument is presented elsewhere^10^.

To assess if mean habitat overlap values were different from those expected by chance, we compared the observed values with the expected means obtained from 1000 simulated null-assemblages. Null-assemblages were simulated using Monte Carlo randomisation algorithms that assign resource use values (in our case, number of individuals from different sampling sites) to each species. The choice of an appropriate model to construct null-assemblages is a critical issue. Four randomisation algorithms have been developed^47^ that differ in whether utilisations are reshuffled or replaced by a random number, and in whether the zeros in the matrix are retained or not. These algorithms are referred to as RA1, RA2, RA3, and RA4. Both retaining/relaxing niche breadths and retaining/reshuffling zeros have implications for the structure of the null community and affect the power of the test^48^. Theoretical and empirical analyses of these algorithms^49, 50^ led to the conclusion that the RA3 is the best existing algorithm for use in resource overlap null models. This algorithm tests for community structure by retaining niche breadth (i.e. the amount of specialisation) for each species (simulated specialisation equal to the observed value), but reshuffles zero states (i.e. by randomly varying the particular resources that were used), thus destroying the guild structure manifested by the zero structure of the resource utilisation matrix.

However, the RA3 algorithm tends to overestimate niche overlap if the equiprobability assumption is not met, because more abundant resources will be used by all species even if niche segregation occurs. Thus, we used, for comparative purposes, also the RA2 algorithm, which tests for structure in the generalist–specialist nature of the resource utilisation matrix by conserving guild structure (zero states are retained, thus preventing species that did not use a certain resource in the field from doing so in simulations), but relaxes niche breadth (thus assuming a random equiprobable specialisation)^48^.

### Between-year differences in niche overlap

To investigate between-year differences in niche overlap, we considered two types of niche overlap values: pairwise overlap *O* values (i.e. *O*_*jk*_ values as given by Pianka’s index, hereafter simply *O*) and species mean overlap (indicated as *O*_sp_). For a given species *j*, the *O*_sp_ value was calculated as the average of the pairwise *O* values of all pairs in which species *j* was involved (for example, in a community with four species, say *j*-*m*, the *O*_sp_ value of the species *j* is the average of the pairwise values *O*_*j,k*_, *O*_*j,l*_, and *O*_*j,m*_).

We analysed between-year differences in *O* and *O*_sp_ values using repeated-measures ANOVAs (rANOVAs) with Type III SS. In these rANOVAs, the repeated-measure factor, i.e. the within-subjects factor, was time (i.e. the three sampling years), while the dependent quantitative variable on which each subject was measured was, alternatively, *O* or *O*_*sp*_. Species-pairs were grouped into three types: pairs in which both species were stygobites (s,s), pairs in which one species was a stygobite, but the other was not (s,n), and pairs in which both species were non-stygobites (n,n). The type of pair was introduced as a between-groups factor in the rANOVA of *O*. Only species pairs present in all three years were considered, which led to the exclusion of four species. The type of species (stygobite, s or non-stygobyte, n) was introduced as a between-groups factor in the rANOVA of *O*_*sp*_. Although only species present in all three years were considered, *O*_sp_ values were calculated, for each year, using all pairwise values of that year. Post-hoc comparisons between groups were performed using Bonferroni’s tests. Analyses were done using the R package ‘ez’^51^. Significance threshold was set at *P* = 0.05 for all tests. Preliminary Kolmogorov-Smirnov’s tests, q-q plots and Levene’s tests indicated no substantial deviations from normality and homoscedasticity, thus no transformation was applied and parametric analyses were performed.

### Species co-occurrence

We also calculated species segregation by using Stone and Robert’s^52^ C-score. The C-score for a species pair *jk* is calculated as:

$${{\rm{C}}}_{jk}=({{\rm{R}}}_{j}-{\rm{SS}})({{\rm{R}}}_{k}-{\rm{SS}})$$

where R_*j*_ is the row total (number of presences) for species *j*, R_*k*_ is the row total for species *k*, and SS is the number of samples that contain both *j* and *k*. Thus, for any particular species pair, the C-score is a numerical index that ranges from a minimum of 0 (maximally aggregated) to a maximum of R_*j*_R_*k*_ (maximally segregated with no shared samples). The matrix-wide C-score is an average of all the pairwise values of C-score for different species, so it reflects both positively and negatively associated species pairs. We were not interested in establishing whether the matrix had an average C-score significantly different from what can be expected from a null-model (which is a very disputed issue^53–56^). Rather, we used average C-scores only as a descriptive tool to compare randomness across years, with higher average C-scores indicating a lower randomness, i.e. a greater likelihood that the distribution of one species has been directly affected by the presence of other species. While niche overlap analysis uses a data matrix with species- and site-specific observed frequencies, the C-score searches for a non-random structure in species assemblages by using a presence/absence data matrix^48^.

To investigate between-year differences in C-score, we used rANOVAs with Type III SS, where the dependent quantitative variable on which each subject was measured was the pairwise C-score. We tested the C-scores calculated for all species simultaneously, and for stygobites and non-stygobites separately.

## Electronic supplementary material

Supplementary Information
