# Supplementary material for: Earthquake-Related Changes in Species Spatial Niche Overlaps in Spring Communities
Source: Sci Rep. 2017 Mar 27;7:443. doi: 10.1038/s41598-017-00592-z (PMC5428671; doi:10.1038/s41598-017-00592-z)
Supplement: Supplementary file 1 — Supplementary Information [file 41598_2017_592_MOESM1_ESM.pdf]

Earthquake-Related Changes in Species Spatial Niche Overlaps in Spring Communities

Simone Fattorini[1,2], Paola Lombardo[3], Barbara Fiasca[1], Alessia Di Cioccio[1], Tiziana Di Lorenzo[4], Diana M. P. Galassi[1]*

[1]Department of Life, Health and Environmental Sciences, University of L'Aquila, L'Aquila (Italy). [2]CE3C – Centre for Ecology, Evolution and Environmental Changes/Azorean Biodiversity Group and University of Azores, Angra do Heroísmo (Portugal). [3]Limno Consulting, Rome (Italy). [4]Istituto per lo Studio degli Ecosistemi, ISE-CNR, Sesto Fiorentino, Florence (Italy).

*corresponding author: simone.fattorini@univaq.it

Supplementary Information

Table S1.

Number of individuals found at the TS sampling stations (TS1 through TS8) in 1997.

Number of individuals as totals found at each site during a complete hydrological year (n=32).

Species name	Dependence on groundwater*	Sampled springs							
		TS1	TS2	TS3	TS4	TS5	TS6	TS7	TS8
Attheyella crassa (Sars G.O., 1863)	nSB	0	2	0	2	2	0	0	2
Bryocamptus echinatus (Mrázek, 1893)	nSB	8	10	19	8	42	0	0	16
Bryocamptus pygmaeus (Sars G.O., 1863)	nSB	0	0	57	2	1	1	0	2
Bryocamptus typhlops (Mrázek, 1893)	nSB	3	0	1	1	30	2	4	7
Bryocamptus zschokkei (Schmeil, 1893)	nSB	0	2	3	7	0	0	1	3
Diacyclops paolae Pesce & Galassi, 1987	SB	1	1	133	10	6	0	9	18
Diacyclops italianus (Kiefer, 1931)	SB	1	0	1	0	0	0	0	0
Elaphoidella mabelae Galassi & Pesce, 1991	SB	0	0	0	0	8	0	0	10
Epactophanes richardi Mrázek, 1893	nSB	0	0	0	0	0	0	1	0
Eucyclops serrulatus (Fischer, 1851)	nSB	0	2	8	1	4	0	0	0
Moraria poppei meridionalis Chappuis, 1929	nSB	0	0	0	0	0	0	6	0
Moraria varica (Graeter, 1911)	nSB	0	0	0	1	0	0	0	0
Nitokra hibernica (Brady, 1880)	nSB	0	0	1	1	0	0	0	0
Nitocrella kunzi Galassi & De Laurentiis, 1997	SB	0	1	0	0	0	2	6	0
Nitocrella pescei Galassi & De Laurentiis, 1997	SB	145	31	13	6	0	20	21	91
Paracyclops fimbriatus (Fischer, 1853)	nSB	0	3	7	0	2	0	2	34
Stammericaris lorenzae (Pesce, Galassi & Cottarelli, 1995)	SB	0	1	2	10	13	1	6	2
Pseudectinosoma reductum Galassi & De Laurentiis, 1997	SB	0	0	1	0	0	0	0	0
Pesceus schmeili (Mrázek, 1893)	nSB	0	2	0	60	21	0	15	0
Parapseudoleptomesochra italica Pesce & Petkovski, 1980	SB	0	0	5	1	0	0	0	2
Simplicaris lethaea Galassi & De Laurentiis, 2004	SB	0	0	0	0	1	4	0	0

*nSB indicates non-stygobiotic species, SB indicates stygobiotic species

Table S2.

Number of individuals found at the TS sampling stations (TS1 through TS8) in 2005.

Number of individuals as totals found at each site during a complete hydrological year (n=32).

Species name	Dependence on groundwater*	Sampled springs							
		TS1	TS2	TS3	TS4	TS5	TS6	TS7	TS8
Attheyella crassa (Sars G.O., 1863)	nSB	1	0	13	1	9	0	23	7
Bryocamptus echinatus (Mrázek, 1893)	nSB	3	34	31	12	52	2	17	22
Bryocamptus minutus (Claus, 1863)	nSB	0	0	0	0	0	0	0	4
Bryocamptus pygmaeus (Sars G.O., 1863)	nSB	4	0	18	0	11	14	2	0
Bryocamptus typhlops (Mrázek, 1893)	nSB	1	2	140	1	110	0	30	0
Bryocamptus zschokkei (Schmeil, 1893)	nSB	0	0	4	1	12	0	1	0
Diacyclops paolae Pesce & Galassi, 1987	SB	3	0	18	2	9	1	48	12
Elaphoidella mabelae Galassi & Pesce, 1991	SB	0	0	4	0	3	0	36	0
Epactophanes richardi Mrázek, 1893	nSB	0	0	0	1	0	0	3	0
Eucyclops serrulatus (Fischer, 1851)	nSB	3	0	2	4	13	0	9	0
Moraria poppei meridionalis Chappuis, 1929	nSB	1	0	3	3	18	0	437	0
Moraria varica (Graeter, 1911)	nSB	0	1	1	0	1	0	0	0
Nitokra hibernica (Brady, 1880)	nSB	0	1	0	0	5	0	0	0
Nitocrella kunzi Galassi & De Laurentiis, 1997	SB	0	1	1	0	0	0	0	0
Nitocrella pescei Galassi & De Laurentiis, 1997	SB	54	151	41	62	3	99	3	185
Paracyclops fimbriatus (Fischer, 1853)	nSB	1	0	1	0	5	0	6	17
Stammericaris lorenzae (Pesce, Galassi & Cottarelli, 1995)	SB	0	14	6	16	16	17	147	0
Pseudectinosoma reductum Galassi & De Laurentiis, 1997	SB	0	0	0	2	0	0	0	0
Pesceus schmeili (Mrázek, 1893)	nSB	2	7	17	38	274	0	333	0
Parapseudoleptomesochra italica Pesce & Petkovski, 1980	SB	4	0	2	0	0	0	0	0
Simplicaris lethaea Galassi & De Laurentiis, 2004	SB	0	0	0	0	0	1	0	0

*nSB indicates non-stygobiotic species, SB indicates stygobiotic species

Table S3.

Number of individuals found at the TS sampling stations (TS1 through TS8) in 2012.

Number of individuals as totals found at each site during a complete hydrological year (n=32).

Species name	Dependence on groundwater*	Sampled springs							
		TS1	TS2	TS3	TS4	TS5	TS6	TS7	TS8
Attheyella crassa (Sars G.O., 1863)	nSB	0	14	26	2	0	0	0	0
Bryocamptus echinatus (Mrázek, 1893)	nSB	2	96	85	26	3	10	2	3
Bryocamptus pygmaeus (Sars G.O., 1863)	nSB	0	0	9	3	0	3	1	0
Bryocamptus typhlops (Mrázek, 1893)	nSB	0	58	71	54	0	0	0	0
Bryocamptus zschokkei (Schmeil, 1893)	nSB	0	1	23	1	0	1	7	0
Diacyclops paolae Pesce & Galassi, 1987	SB	0	4	16	6	4	1	1	1
Elaphoidella mabelae Galassi & Pesce, 1991	SB	1	22	11	6	1	0	0	1
Eucyclops serrulatus (Fischer, 1851)	nSB	0	59	13	3	0	5	1	1
Moraria poppei meridionalis Chappuis, 1929	nSB	0	2	26	9	0	0	0	0
Moraria varica (Graeter, 1911)	nSB	0	0	2	1	0	0	0	0
Nitokra hibernica (Brady, 1880)	nSB	0	4	9	5	3	2	0	0
Nitocrella kunzi Galassi & De Laurentiis, 1997	SB	0	0	0	2	0	0	0	0
Nitocrella pescei Galassi & De Laurentiis, 1997	SB	9	28	7	24	0	2	5	11
Paracyclops fimbriatus (Fischer, 1853)	nSB	0	1	1	0	2	0	0	0
Stammericaris lorenzae (Pesce, Galassi & Cottarelli, 1995)	SB	0	5	4	5	3	0	3	0
Pesceus schmeili (Mrázek, 1893)	nSB	0	38	16	17	0	0	1	0
Parapseudoleptomesochra italica Pesce & Petkovski, 1980	SB	0	2	1	0	0	0	0	0
Simplicaris lethaea Galassi & De Laurentiis, 2004	SB	0	0	0	0	0	1	1	0

*nSB indicates non-stygobiotic species, SB indicates stygobiotic species
